# Analysis of the Emotional Exhaustion Derived From Techno-Stress in the Next Generation of Qualified Employees

**DOI:** 10.3389/fpsyg.2022.792606

**Published:** 2022-02-08

**Authors:** María Buenadicha-Mateos, María Isabel Sánchez-Hernández, Óscar Rodrigo González-López

**Affiliations:** University of Extremadura, Badajoz, Spain

**Keywords:** suffering, technostress, perceived stress, interpersonal conflicts, emotional exhaustion, higher education

## Abstract

This study analyses the emotional exhaustion of students inhigher education, derived from the extremely technology-relatedstrain associated to the current COVID-19 pandemic in a conservation of resources’ approach. Technostress, as source of emotional exhaustion, was investigated in a sample of 333 students in a medium size public university in Spain. Data was collected in May 2020, during the COVID lockdown. After literature review, a structural model was developed, linking technostress with emotional exhaustion. Results confirm the expected cause-effect relationships. In addition, the study reveals two mediator variables that must be considered when managing students′ suffering, perceived stress and intrapersonal conflicts. This study contributes to the academic literature in the field of managing and mitigating suffering. They do so by providing both new knowledge and empirical evidence on the effects of technostress in the new generations that will soon join the working life.

## Introduction

There are numerous positive aspects that can be highlighted in the use of information and communications technology (ICT) in both the personal and the occupational sphere. Nonetheless, they can also generate new psychosocial risks which cause suffering. That is something that should not be ignored, and in fact, it must be adequately studied. It is especially interesting to address specific studies on the impact that the use of ICT in university students has, especially considering that these students will be the new cohorts of qualified employees in the near future. In addition, they count with a more integrated use of ICT in their daily lives and needing to use them to carry out their educational activities. Furthermore, as Arnett (2000) has argued, university students experience critical transitions in their youth, which are mainly characterized by change, confusion and exploration. This way, both their actions, and decisions made during this period of time can have lasting ramifications, making their consequences more relevant than for other groups.

It has been pointed out that the overexposure of ICT in young people is an understudied phenomenon (Lutz et al., 2014). That may have been partly due to the belief that, as they are digital natives, they are able to assume the risks associated with technology more easily than other subjects(Prensky, 2001). It seems that university students are being assumed to be highly tech-savvy, and therefore techno-stress free (Wang et al., 2020).

But, when talking about university students, it must be considered that although it is important to take into account the experience they have with ICT, the possible influence of the intensity of use must also be assessed. For instance, even before the COVID-19 pandemic, it was quantified that two thirds of students would rather leaving their smartphones on during night-time (Vorderer et al., 2016). It may be thought that the high frequency of ICT used in students belongs to their personal communication sphere. Nevertheless, it should also be considered that the use of ICT in their academic life it is increasing, as a result of universities aiming to achieve academic success in students. In fact, universities around the world have continuously increased investment in the use of technology to evolve mainstream education, and technology-enhanced learning is becoming increasingly important in higher education (Dunn and Kennedy, 2019). As a consequence, university students in this environment of intensified use of ICT, can develop a worrying emotional exhaustion that, unfortunately, contributes to the deterioration of their well-being (Cotten, 2008), quality of life (Bradley, 2017), and their mental health (Mofatteh, 2021).

If emotional exhaustion itself already requires specific studies, the quarantine imposed by governments to prevent the spread of COVID-19 has led to an intensification of the use of ICT in the population, which may have aggravated the situation. Due to the compulsory closure of educational institutions, students were forced to use these ICT tools intensively in order to continue studying at a distance (online teaching and learning processes), suddenly and without planning or specific preparation. For instance, in the month of March 2020, the government of Spain during the COVID-19 pandemic eliminated face-to-face classes in universities, forcing all teachers to continue their classes remotely.

The impact of the pandemic on mental health and education is expected to be significant (Sahu, 2020). It is relevant to consider that more than half of the students (54.9%) have indicated that distance learning was their greatest concern during this pandemic time (Al-Tammemi et al., 2020). The mental health of university students must be controlled during epidemics (Cao et al., 2020) since, during these periods, certain social components (such as the elderly, children, healthcare workers, infected patients, patients with pre-existing psychiatric conditions, and students) are at increased risk of experiencing a significant degree of both psychological pressure and stress, compared to other people (Ho et al., 2020).

In this context, we must pay attention to the fact that some of the negative effects of quarantines are techno-stress and emotional fatigue. On the one hand, there’s techno-stress, since the irregular situation of the pandemic could have caused an increase in negative predictors of technophobia even in people who previously adopted technology (Daruwala, 2020), such as university students. On the other hand, emotional fatigue is one of the negatives psychological effects from quarantines (Maunder et al., 2003).

This study tries to contribute to the knowledge of the emotional fatigue of university students, as well as assessing its connection with techno-stress: although ICT has provided students the tools to successfully go on with both their social and academic lives, it may also be a source of techno-stress and suffering, having negative effects on students′ well-being.

## Theoretical Background and Hypotheses Development

### Theoretical Underpinning of the Study

The Conservation of Resources theory (COR), proposed as a theory of stress and motivation (Westman et al., 2005), is the integrative framework assumed in this study. According to COR, human beings are motivated to accomplish and safeguard resources, and acquire new ones (Hobfoll, 1989, 2001). Moreover, this theory also postulates that stress appears when facing potential or actual loss, or the lack of earned resources. COR theory is considered a major theory in different fields such as leadership (Fatima et al., 2018); burnout (Arnold et al., 2015); and more specifically, student burnout (Alarcon et al., 2011); exploring the transition from study to work (Robins et al., 2018) within positive psychology (Hobfoll, 2011; Carmona-Halty et al., 2019).

The COVID pandemic has created a hostile environment in higher educational contexts where resources have been threatened. Losses of tangibles resources such as physical space, and intangible resources such as self-esteem or self-efficacy (Halbesleben et al., 2014). The last ones have been proven to be more salient to students than potential gains. As technological demands increase to follow the courses in pandemic times, and traditional resources have been lost, students can become exhausted. Based on COR theory, we argue that students put up with technostress in COVID times as they feel resource deficiencies and losses in their new normality, flowing into emotional exhaustion. Their previous intra personal conflicts and perceived stress will mediate the relationship, as discussed bellows.

### Emotional Exhaustion as a Specific Dimension of Burnout

Burnout has been gaining importance in recent years. Despite being firstly focused on professional services with human relations (such as teachers, doctors, etc.), it is now no longer restricted to these professions, and has a wide scope of application that includes burnout jobs in students (Maunder et al., 2003; Navarro-Abal et al., 2018; Worly et al., 2019; West et al., 2020). In fact, the concern about student burnout is not new. At this respect, Law (2007) pointed out that student burnout levels were extreme compared to those in traditional high-burnout occupations.

Emotional exhaustion can be used as an indicator of students’ emotional well-being (Devine and Hunter, 2016) and to evaluate their quality of life (Li et al., 2018). It is also a very important dimension of burnout. Emotional exhaustion can be described as a chronic state of emotional and physical fatigue (Maslach et al., 2001). It is related to feelings of exhaustion from emotional sources (Parker and Salmela-Aro, 2011). It assumes an erosion of satisfaction with life (Hakanen and Schaufeli, 2012), and can basically be understood like feeling exhausted and therefore not interested in one’s occupation (Daumiller et al., 2021). Moreover, it can even make people feel physically fatigued (Wright and Cropanzano, 1998). In general, the toxic consequences of emotional exhaustion include mental and physical health problems, deterioration of social and family relationships, and, at the professional level, dysfunctional outcomes in the individual’s relationship with their supervisor (Soderlund, 2017).

From all the above, it can be deduced that emotional exhaustion is a very relevant variable to which we must pay attention, given the forced and non-progressive intensification of the use of ICT by students due to the prevention measures of contagion of COVID-19.

### Techno-Stress at the University Level

The pioneer in awakening interest in the term “techno-stress” is Brod, in the 80s. He defines it basically as an adaptive disease that has its origin in the lack of ability to deal with new technologies in a healthy way (Brod, 1984). We must consider that techno-stress is not limited to high-tech or business work contexts (Wang et al., 2020). Techno-stress studies are important when considering an aging workforce with longer careers, in which growth technology can increase vulnerability to stress and affect the health and well-being of professionals (Chaves et al., 2016). However, it would be a serious mistake to leave aside other population groups, in part guided because they are presumed to dominate prior technology. This is the case of university students.

The pandemic scenario has generated stress problems for students, driven among other reasons by the intense introduction of distance learning and uncertainty in universities (Agnew et al., 2019). Some years ago issues such as information overload, and techno-stress, were highlighted as important challenges of the information age (Lutz et al., 2014). Still, in the current context, their importance has only made it grow, partly due to the fact that the level of ICT use is a significant predictor of techno-stress levels (Coppari et al., 2017). As an example of its importance, in Lindén et al. (2018)’s work it is shown that techno-stress is one of the two stressors that most frequently appear (along with task overload). Nonetheless, at the moment, there has been a dearth of research on this topic in the field of education, particularly in higher education (Wang et al., 2020). Although some studies are contributing to understand techno-stress in university teachers (Penado Abilleira et al., 2021), new works are also needed at the student level (Penado Abilleira et al., 2020; Upadhyaya, 2021).

### Techno-Stress Linkages to Emotional Exhaustion

It should be considered that, despite the great benefits that the intensification in the use of technology in learning in higher education can offer, it can also cause techno-stress (Wang et al., 2020). In many cases, it has been assumed that university students, belonging to a generation which is very familiar with ICT, are not so predisposed to suffer these risks. It is necessary to address the study of techno-stress in these students since, in addition to their personal sphere, they have a greater exposure to ICT than students at other school levels. This is due to the widespread adoption of technology-enhanced learning in higher education (Dunn and Kennedy, 2019).

According to Llorens et al. (2011), techno-stress is a multidimensional phenomenon with four dimensions: anxiety, fatigue, skepticism and ineffectiveness, which correspond to three different aspects: cognitive (ineffectiveness), attitudinal (skepticism) and affective (anxiety and fatigue). It is interesting to approach techno-stress through its dimensions, which will allow a more complete and coherent understanding.

Techno-stress is a source of problems that can affect different spheres of the sufferer. The classifications are varied. They can be problems of the individual, group and organizational sphere (Salanova et al., 1999); physical, social and emotional (de Souza and Cappellozza, 2018); problems in professional and private life (La Torre et al., 2019); individual, group and professional (González-López et al., 2021), among others. Nonetheless, as it can be seen, the effects on the subject itself are shown in every group. In fact, if we consider the negative effects of techno-stress as individual, group or professional, it is the individual negative effects of techno-stress that is the most important, although González-López et al. (2021) have demonstrated that these three levels are related in a statistically significant way. Within its individual effects, techno-stress is often associated with the appearance of psychological and behavioral disorders (La Torre et al., 2019). Still, in this case, it is especially relevant that techno-stress can cause burnout (Salanova et al., 2002; Chaves et al., 2016; Wang et al., 2020).

Recent studies corroborate that two specific techno-stressors (techno-overload and techno-invasion) stimulate emotional exhaustion in a contextualized study in childcare (Bauwens et al., 2021). In a specific context of students, the study of Wang et al. (2020) shows that the dimensions of techno-stress have a positive relationship with burnout. Thus, it is interesting to investigate whether the stress generated by technology could be generating emotional exhaustion in students. In fact, there are authors who speak specifically of digital burnout, within the context of COVID-19, related to hyper-connection to smartphones, laptops and tablets that leaves us susceptible to exhaustion (Sharma et al., 2020).

All of the above, plus some results such as those from the study by Silva et al. (2016) where positive correlations are shown between all the dimensions of techno-stress and emotional exhaustion (Chaves et al., 2016) outside of this pandemic context, serve as the basis for exploring this case in university students in the context of a pandemic. This allows us to posit the first hypothesis of the study:

H1: Techno-stress is direct and significantly related to the emotional exhaustion of university students.

### The Mediation Role of Perceived Stress and Intrapersonal Conflicts

It is worth exploring the relationships of stress produced by technology, perceived stress and intrapersonal conflicts such as digital attachment. Along these lines, it is known that the risk of addiction to smartphones has a positive relationship with perceived stress (Samaha and Hawi, 2016). There is also a strong relationship between perceived stress, mental health and problems with life academic with burnout syndrome (Dimitriu et al., 2020; Zaed et al., 2020).

Perceived stress is a measure that collects the perception of particular stress, independently of external environmental stressors. Considering that the level of perceived stress is inversely correlated with the quality of life and well-being of persons (Ribeiro et al., 2018), there is no doubt that nowadays stress is a problem with serious physical and psychological consequences. The fact that, when students are asked what are the most frequent negative feelings they experience in their lives, these are fatigue, stress and boredom (Moeller et al., 2020), makes it even more tragic. That fatigue and stress are undoubtedly particularly worrisome at a vital stage where the foundations of their professional future are being laid.

There are studies where a comparison of perceived stress before and during the pandemic is made, and it is shown that stress has increased (Elmer et al., 2020; Özdin and Bayrak Özdin, 2020; Son et al., 2020). In addition, students are aware of this fact. For example, in the study of Son et al. (2020), the majority of students (71%) indicated an increase in perceived stress during the COVID-19 pandemic compared to the pre-pandemic period. Obviously, the pandemic has generated tensions in students for multiple causes, and the abrupt introduction of distance learning and uncertainty in universities are of great relevance (Agnew et al., 2019). For all of the above, the following hypothesis is posited.

H2: The relationship between techno-stress and emotional exhaustion is mediated by perceived stress.

While some of the key factors for student learning in times of pandemic have been good Internet connectivity and the ability to easily relate and communicate with teachers (Katz et al., 2021), the dependence of communication on the online environment may have caused some problems for the students. Users can easily become dependent on (or even addicted to) ICT, providing streams of hedonic gratification and short-term stimulation (Eyal, 2014). There could also be an over-identification with the technology that would tend to dissolve the boundaries of person-machine interaction and create dependence on technology (Sayers et al., 2021).

There is a growing acceptance of the relationship between obsessive, compulsive and excessive use of digital media and the well-being of users (Cham et al., 2019). This justifies the current need for determine the reasons for people’s excessive commitment and the subsequent impact on indicators of well-being and functional deficiencies (Tandon et al., 2021).

It is convenient to approach this problem from a gradual perspective, and bear in mind that there are different intensities and levels that must be considered. Thus, in the literature, we find terms such as dependency, problematic attachment, excessive commitment or addiction, among others. Some authors have even pointed out that the term “internet addiction” has become obsolete (Carbonell et al., 2012). Dependence does not necessarily indicate obsessional behavior, or typical addiction symptoms (Turel et al., 2011). For example, Altuwairiqi et al. (2019) pointed out that an obsessive and excessive use leads to negative impacts on one’s life, such as what they call a problematic attachment.

The truth is that users who spend greater amounts of time on the internet are more likely to present a connected behavior controlled by negative reinforces, a high degree of excitement when they are online, loss of control, changes in health habits, and interference at the social, family, academic or work level (Muñoz-Rivas et al., 2010). Moreover, the omnipresence of technology during the lockdown could have caused an increase in the behaviors of addiction to the internet or digital devices, and this can be identified as a consequence of techno-stress (Molino et al., 2020).

Regarding burnout, there are studies that indicate that a higher level of burnout in students is significantly related to a higher level of problematic internet use (Tomaszek and Muchacka-Cymerman, 2020), and they show a positive and significant relationship between the mobile use and psychological disorders and academic burnout (Noruzi Kuhdasht et al., 2018). Based on all the above, it is possible to posit the following hypothesis.

H3: The relationship between techno-stress and emotional exhaustion is mediated by the level of intrapersonal conflicts of students.

### Theoretical Model

**FIGURE 1:**
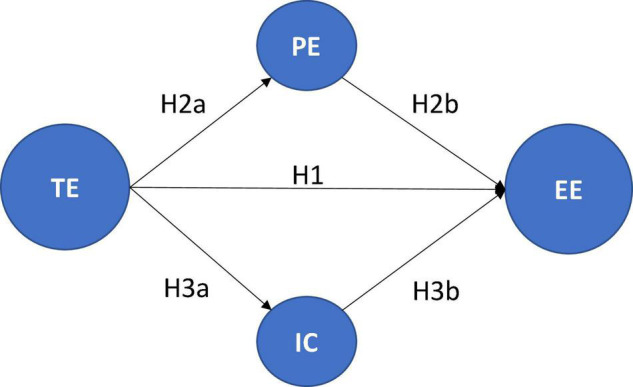
Theoretical model.

The theoretical framework previously developed allow us to define the theoretical model shown in Figure 1, where TE is techno-stress, PE is perceived stress, IC is interpersonal conflicts and EE is emotional exhaustion. Hypotheses are H1, H2 (divided in H2a and H2b), and H3 (divided in H3a and H3b).

## Methodological Procedure

### Method

Bearing in mind the exploratory nature of this study, Structural Equation Modelling (SEM) and Partial Least Squares (PLS) have been selected following Hair et al. (2021). PLS-SEM also allow us a causal-predictive approach that emphasizes the prediction of emotional exhaustion by the other variables in the model, specifically techno-stress. SmartPLS v.3.3.2 by Ringle et al. (2015) was used for performing the multivariate required analyses of the measurement model and the structural model, both designed to provide the causal required explanations. Some recent records applying SEM on the field do exist (Penado Abilleira et al., 2020, 2021), and also SEM-PLS (Shi et al., 2020; González-López et al., 2021).

### Instrument

A survey instrument was developed to measure students’ techno-stress and related variables in the model. The questionnaire was designed using dimensions and items from previously published academic studies on the topic. Techno-stress has been considered as a second order construct with 16 items distributed in four dimensions (anxiety, fatigue, skepticism, and ineffectiveness), which is recommended for workers but also for pre-occupational samples (Llorens et al., 2011). Perceived stress has been approached by six items trying to catch the perceptions of students, regardless the context, as indicated by Kamarck et al. (1983). Intrapersonal conflicts are measured through three items coming from the Internet Related Experiences Questionnaire (IREQ) by Beranuy et al. (2009) evaluating the level of dependence and evasion of internet users. Finally, seven items were used for approaching the emotional exhaustion of students. The items come from the Maslach burnout inventory (Maslach and Jackson, 1986), with a specific adaptation to the context of the study, changing workers by students, and to work for to study. Table 1 presents the data collection instrument in full in order to allow the study to be replicated.

**TABLE 1:** Instrument.

Techno-stress (TE)	
Anxiety	I feel tense and anxious when working with technology. I doubt myself when using technology afraid of making mistakes. It scares me to think that I can destroy a lot of information due to the incorrect use of technology. Working with technology makes me feel uncomfortable, irritable and impatient.
Fatigue	I find it hard to relax after a day of using technology. When I finish working with technology, I feel exhausted. I’m so tired of working with technology that I can’t do anything else. It is difficult to concentrate after working with technologies.
Skepticism	I am less and less interested in technology. I feel less involved with the use of technology. I am more cynical about the contribution of technologies. I doubt the meaning of working with technologies.
Ineffectiveness	I feel that I am inefficient when using technology. I find it difficult to work with technology. People say that I am inefficient when I use technology. I’m not sure I’m completing tasks well when using technology.
Perceived stress (PS)	I have felt unable to control the important things in your life. I have felt nervous or stressed. I have been confident about my ability to handle my personal problems (reverse). I have felt that things are going well for me (reverse). I felt like I had everything under control (reverse). I have felt that the difficulties accumulate so much that I cannot overcome them.
Intrapersonal conflics (IC)	I plan my next Internet connection very frequently. I get irritated when someone bothers me while I’m online. I find it easier or more comfortable to interact with people online than in person.
Emotional exahustion (EE)	I feel emotionally exhausted because of my studies. I feel tired at the end of the day. When I get up in the morning and face another day, I feel fatigued. I feel that studying all day takes a lot of effort and makes me tired. I feel burned out by my studies. I feel frustrated about my studies. I feel finished in my studies, at the limit of my possibilities.

### Sample and Procedure

Techno-stress and its relationship with the other variables of the theoretical model has been investigated in a sample of 333 students in a medium size public university in Spain. Data was collected in May 2020, during COVID-19 lockdown as is explained as follows.

To carry out this work, a self-administered questionnaire designed *ad hoc* has been used: Google Forms from Google Drive. All the measures were derived from previously validated scales, as explained before, and were administered in Spanish after a translation procedure. The virtual campus was used to send the students the address of the questionnaire, as well as an Access QR code. They could see it in the activities part of the subject’s platform, but they also received a message in their email.

The questionnaires were completed by the students during May 2020, in a context of lockdown, and follow-up of the distance subject imposed for the whole country since March 16, 2020 (so when taking the questionnaire, the students had already been 2 months of forced digital intensification and they were close to finishing the contents and being evaluated in the different subjects).

In the message that the students received, the objectives of the study were explained. The aspects related to the fact that participation was totally voluntary, that the information would be totally confidential, and that the information would be totally anonymous were developed. They were informed that the data would not allow their personal identification, thus complying with the ethical standards, recommendations and regulation of personal data established for this type of work. In addition, students had to explicitly give their consent to participate in the study, in order to use this information for the sole and exclusive purpose of research on emotional exhaustion. To this end, a mandatory question was included at the beginning of the questionnaire which, if not answered affirmatively, it blocked the questionnaire.

The collaborating students were motivated with the idea that their contribution would allow detecting factors that were affecting their well-being, and would allow them to help develop actions aimed at mitigating suffering in students. With this approach, they were involved in a higher order of improvement of the university community. During data collection, the students had the advice of a researcher through an email address.

## Results and Findings

**FIGURE 2:**
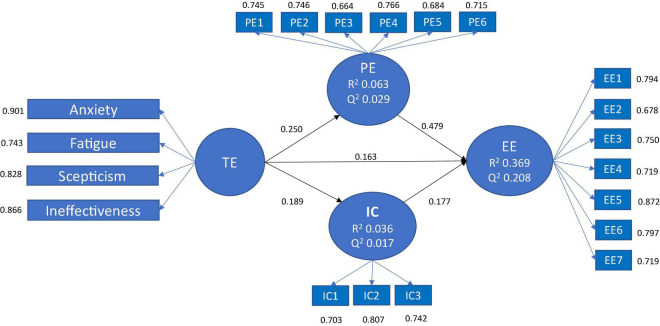
Main results.

The main results are shown in Figure 2 (mainly related to the measurement model), and Table 2 (for hypotheses testing validating the structural model after bootstrapping procedure with 5,000 resamples).

**TABLE 2:** Hypotheses testing.

Hypothesis	Path coefficient (original)	Path coefficient (sample)	St. Error	Confidence interval [2.5–97.5]%	*t*-statistics	Significant (*p* < 0.05)
**Total effects**						
H1: TE → EE	0.163	0.163	0.051	0.065–0.262	3.179	0.001
H2a: TE → PE	0.250	0.256	0.055	0.144–0.360	4.538	0.000
H2b: PE → EE	0.497	0.481	0.045	0.390–0.565	10.733	0.000
H3a: TE → IC	0.189	0.195	0.057	0.080–0.304	3.327	0.001
H3b: IC → EE	0.177	0.180	0.049	0.083–0.276	3.595	0.000
**Mediation effects**						
H2: TE → PE → EE	0.120	0.123	0.029	0.067–0.182	4.127	0.000
H3: TE → IC → EE	0.033	0.034	0.013	0.012–0.062	2.639	0.008

Related to the measurement model, reliability was assessed by examining individual loads of the items with their respective constructs (≥0.66 was accepted). The Cronbach’s alpha coefficient was used as an index of reliability of the latent variables, ranging from 0.88 for emotional exhaustion, to the slightly low value of 0.62 for interpersonal conflicts. Composite reliability was also calculated showing acceptable values of 0,903 for techno-stress, 0.907 for emotional exhaustion, 0.866 for perceived stress, and 0.795 for intrapersonal conflicts. The convergent validity of all constructs in the model was evaluated through the average variance extracted (AVE) and was accepted in all cases (>0.5) (Hair et al., 2021). The discriminant validity of the latent variables was verified using the Fornell-Larcker criterion and the HTMT ratio (<0.90) (Henseler, 2017).

Moving to assess the structural model, the explained variance (R^2^) of the endogenous latent variables and the regression coefficients were showed in Figure 2. The *p*-values of the regression coefficients (test t) are shown in Table 2, and are used as indicators of the explanatory power of the model, confirming also the mediator role of both perceived stress and intrapersonal conflicts in the relationship between techno-stress and the emotional exhaustion of students.

The overall fit of the model was evaluated using the standardized root mean square residual indicator (SRMR) (Hu and Bentler, 1998). In this study, the SRMR was 0.069, which means that the model fits the empirical data (Henseler et al., 2016). Other fit measure in SEM literature is NFI, the normed fit index by Bentler and Bonett (1980). The closer the NFI to 1, the better fit. The model tested here has obtained a good value of 0.796.

Finally, the predictive relevance of the model was analyzed through the Stone-Geisser test (Q^2^) after a blindfolding procedure. In the model tested, all endogenous constructs fulfill Q^2^ > 0, with values of 0.208, 0,029, and 0.017 for emotional exhaustion, perceived stress, and intrapersonal conflicts, respectively.

The findings confirm the multidimensional nature of technostress among university students. The four dimensions - anxiety, fatigue, skepticism and ineffectiveness – have been confirmed, corroborating techno-stress as second order construct that impacts directly to the emotional exhaustions of students and both, perceived stress and interpersonal conflicts. It has been also demonstrated that these two last constructs have a mediation effect between techno-stress and emotional exhaustion.

## Conclusion

This study contributes to the academic literature in the field of managing and mitigating suffering in the new generations that will soon join the working life. They do so by providing both new knowledge and empirical evidence on the effects of technostress in university students. In this work, the theoretical development of a causal model relating techno-stress and the empirical exhaustion of students in COVID pandemic times, is empirically tested and validated. Results let us confirm the dangerous consequences of ICTs if they are not well managed. In addition, it might be thought that perceived stress and individual conflicts could both be mediators of the effect between techno-stress and students’ emotional exhaustion.

It has been demonstrated in this study that university students are not tech-savvy because they are not techno-stress free. The COR theory suggests that students, as individuals, continuously strive to seek, acquire, and maintain resources. This theoretical framework justifies that students react to the substitution of traditional lectures to on line teaching methods, in which there is the threat of a loss of resources. Technostress and emotional exhaustion will be the reactions until they gain some resources to cope up with the resource losses. Ensuring that students acquire ICT skills and found the required support from their universities (as new resources) may be the solution.

## Implications

Improving the quality of life of students should be one of the main concerns of universities, due to the fact they will be the next generation entering the labor market. It is a matter of preventing their potential future suffering at work. In recent years, the use of ICT in university studies has been intensified and, although the internet and social networks can be excellent tools to maintain a social relationship with classmates, high school friends and family, they can also have negative effects on students’ mental health (Mofatteh, 2021). The issue here is that students have been very vulnerable during the COVID-19 pandemic, and researchers have in fact proven that mental health problems have increased in that period (Aslan et al., 2020).

New studies about techno-stress and related variables such as emotional exhaustion, are essential for its detection, management, prevention, and mental illnesses (Makhubela, 2020). In a context such as the pandemic, the study of perceived stress and intrapersonal conflicts also seems to be especially relevant. In fact, the perception of the impact of COVID-19 on the well-being of students turned out to be a significant predictor of perceived stress (Aslan et al., 2020). Moreover, the strongest relationship between global techno-stress occurs with individual negative consequences (González-López et al., 2021), where intrapersonal conflicts such as digital attachment are framed. Given that fact, new studies must be done to explore whether these individual problems could influence other psychological aspects of students’ life, and condition their access to the labor market with guaranties of personal and professional success.

## Limitations

Regarding the generalization of results, this study has some limitations that must be acknowledged, as other previous studies in the field have done (Penado Abilleira et al., 2020, 2021): (1) the cross-sectional data instead of longitudinal data, (2) the use of self-evaluation questionnaire instead of objective data, and (3) the absence of control variables to validate the instrument in any context. However, we hope that the validated model in the Spanish context will serve to other researchers to replicate the study and shed light on the relationship between techno-stress and emotional exhaustion in university students in pandemic times. In addition, the research team have new planned studies for the near future that will allow to contribute to the field with new insights.

## Data Availability Statement

The raw data supporting the conclusions of this article will be made available by the authors, without undue reservation.

## Ethics Statement

Ethical review and approval was not required for the study on human participants in accordance with the local legislation and institutional requirements. The patients/participants provided their written informed consent to participate in this study.

## Author Contributions

MB-M performed the data collection, searched and reviewed the study, wrote the manuscript and supervised the research process. MS-H analyzed the data, wrote and critically reviewed the manuscript. ÓG-L performed the data collection, and critically reviewed the manuscript. All authors contributed to data interpretation and approved the final version of the manuscript for submission.

## Conflict of Interest

The authors declare that the research was conducted in the absence of any commercial or financial relationships that could be construed as a potential conflict of interest.

## Publisher’s Note

All claims expressed in this article are solely those of the authors and do not necessarily represent those of their affiliated organizations, or those of the publisher, the editors and the reviewers. Any product that may be evaluated in this article, or claim that may be made by its manufacturer, is not guaranteed or endorsed by the publisher.
